# Supplementary material for: Diagnostic Challenges in Extrapulmonary Tuberculosis: A Single-Center Experience in a High-Resource Setting at a German Tertiary Care Center
Source: Infect Dis Rep. 2025 Apr 23;17(3):39. doi: 10.3390/idr17030039 (PMC12101312; doi:10.3390/idr17030039)
Supplement: Supplementary file 1 [file idr-17-00039-s001.zip › Country of Origin_Supplemental table 1.pdf]

	n	%
Germany	50	25.8
Unknown	15	7.7
Pakistan	11	5.7
Syria	10	5.2
Ukraine	9	4.6
Eritrea	9	4.6
Turkey	6	3.1
Russia	5	2.6
Nigeria	5	2.6
Guinea	5	2.6
Afghanistan	4	2.1
Romania	4	2.1
Sri Lanka	4	2.1
Azerbaijan	4	2.1
India	3	1.5
Morocco	3	1.5
Iran	3	1.5
Kosovo	3	1.5
Mali	3	1.5
Kazakhstan	3	1.5
Hungary	2	1.0
Iraq	2	1.0
Kyrgyzstan	2	1.0
Nepal	2	1.0
Macedonia	2	1.0
United Kingdom	2	1.0
Tajikistan	2	1.0
Bangladesh	2	1.0
Angola	2	1.0
Algeria	2	1.0
Belgium	1	0.5
Congo	1	0.5
Latvia	1	0.5
Gabon	1	0.5
Brazil	1	0.5
Libya	1	0.5
Sudan	1	0.5
Peru	1	0.5
Lebanon	1	0.5
Ivory Coast	1	0.5
Indonesia	1	0.5
Bulgaria	1	0.5

Albania	1	0.5
Poland	1	0.5
Ethiopia	1	0.5

Supplemental Table S1: Countries of origin for the whole study cohort
