# Supplementary material for: Diagnostic Challenges in Extrapulmonary Tuberculosis: A Single-Center Experience in a High-Resource Setting at a German Tertiary Care Center
Source: Infect Dis Rep. 2025 Apr 23;17(3):39. doi: 10.3390/idr17030039 (PMC12101312; doi:10.3390/idr17030039)
Supplement: Supplementary file 1 [file idr-17-00039-s001.zip › Symptoms Supplemental table 2.pdf]

Supplemental Table S2: Comparison of clinical symptoms between symptomatic patients with isolated pulmonary tuberculosis (n=64) and those with extrapulmonary/disseminated tuberculosis (n=82). Significant differences are highlighted by p-values <0.05.

	Isolated pulmonal tuberculosis (n=64)	Extrapulmonal/disseminated tuberculosis (n=82)	p-value
Thoracic pain	10 (15.6%)	2 (2.4%)	0.005
Abdominal pain	1 (1.6%)	19 (23.2%)	<0.001
Cough	53 (82.8%)	9 (11%)	<0.001
Fever	14 (21.9%)	19 (23.2%)	1
Night sweats	14 (21.9%)	11 (13.4%)	0.19
Headache	0	7 (8.5%)	0.018
Dyspnea	8 (12.5%)	6 (7.3%)	0.4
Hemoptysis	7 (10.9%)	0	0.003
Weight loss	16 (25%)	22 (26.8%)	0.85
Lymph node swelling	0	36 (43.9%)	<0.001
Bone pain	1 (1.6%)	18 (22%)	<0.001
Neurological symptoms	0	3 (3.7%)	0.27
